# Upon the Cure of Popliteal Aneurisms by Digital Compression Suggested by the Numerous Communications to the Society of Surgery

**Published:** 1858-01

**Authors:** A. Verneuil

**Affiliations:** Agrégé of the Faculty of Paris


*Translated from the Gazette Hebdomidaire, by* W. F. Westmoreland, M. D., Professor of Surgery in the Atlanta Medical College.

### ARTICLE II.

*Upon the cure of Popliteal Aneurisms by digital compression suggestedby the numerous communications to the Society of Surgery.* By A. Verneuil, Agrégé of the Faculty of Paris.

Since the publication of the excellent book of our worthy friend M. Broca, the question of the cure of aneurisms has been under consideration, and compression has regained a pre-eminence which it should have never lost. Perhaps we may attribute this, to a certain point to the great noise made against operative Surgery. Still, the Society of Surgery, has recently had the good fortune to receive three observations in which aneurisms was cured by digital compression. We recently announced the communication of M. Vanzetti, of Padone; at the same time, the Society received a communication from M. Michaux, of Louvain, containing a similar fact, which without the detail we give below:

M. Vanzetti, who had seen compression employed at Dublin in 1843, thought to replace the mechanical means there employed by the hands of several assistants who were to succeed each other. In 1846 he made an experiment at the hospital of Kharkoff, Russia, in connection with M. Serebriakoff, Surgeon of this hospital. He instructed the aids himself in regard to the place and manner of making the compression. Compression was continued for two days without effect. The ligature was afterwards resorted to.

Seven years later, (1854) received in his wards at Padone, a mason, about thirty-six years old, good constitution, attacked with popliteal aneurism, well characterized, though of ordinary volume. Pulsations ceased in the tumor upon the compression of the femoral artery. M. Vanzetti determined to try compression, but discouraged by the failure above reported, he employed quite a number of modes of compression with much patience and great inconvenience to the patient.

Before ligating the femoral artery, he wished again to try digital compression, in the hope that, under his immediate inspection he would prove more successful. Several aids, seated and standing, sometimes with one hand and sometimes with both, compressed the artery with moderate force, sufficient, however, to bring in contact the walls of the artery, without any inconvenience to the patient. The place selected for the compression was the middle third of the thigh, thus preventing the obliteration of the profundus.

At the expiration of twelve hours, there was considerable abatement in the pulsation of the tumor. At the end of forty-eight hours, the pulsations had entirely ceased, and no sound could be heard; the compression was removed. The cure was perfect. The tumor in the popliteal region gradually disappeared, and the thigh became straight. The patient has resumed his profession; walks without limping, and experiences no uneasiness in the movements of the knee.

The second observation is still more remarkable. In 1855, a very intelligent officer, 75 years old, consulted M. Vanzetti, in regard to a popliteal aneurism of his right leg. Digital compression was resolved upon, but deferred five days, during which time the patient was to use compression himself, the action of which had been explained to him. The tumor which was small, appeared to have diminished under this very imperfect trial.

The treatment proper was commenced at noon, and intrusted to six intelligent and reliable aids. At six o'clock, M. Vanzetti called to see the progress of the treatment, and found the patient in a sound sleep. To the great surprise of the surgeon, not one of the aids were near him. His surprise was still greater when he was informed that after four hours of the most scrupulous compression, the pulsations, as well as the sounds of the tumor, entirely disappeared. The patient remained at the clinic for one month. He was seen several months afterwards by M. Vanzetti. There was no lameness, and the movements of the leg were free and easy. The aneurismal tumor was converted into a hardened mass the size of a hazel nut. Such are the facts communicated by the surgeon of Padone, and which he has exposed with remarkable clearness. M. Marjolau has also communicated two observations, addressed by M. Michaux, of Louvain, one of the most distinguished foreign surgeons.

The first is a long surgical drama, which commenced by double and intermittent compression, and ended with the amputation of the thigh, necessitated by the mortification of the extremity after the ligation of the femoral artery. The patient was cured. This observation is one of great interest, but as it is not directly to the point we wish to discuss, we will not speak longer about it; the details may be read in the bulletins of the Society.

The following is a brief analysis of the second observation. A man fifty years of age, good constitution, having always enjoyed excellent health, complained for the first time in 1854, of rheumatism in the left thigh. In the month of August, 1856, the patient discovered upon the anterior and internal portion of the thigh, a pulsating tumor situated six centimetres below the crural arch. So rapid was its progress, that by the month of Nov., its transverse diameter was 14 centimetres, and its vertical 11 centimetres. All the signs of aneurism were well marked. By thorough examination of the patient, a general alteration of the arterial system was evident; abnormal sounds towards the centre of the circulatory system, a rude souffle was heard in all the large arteries. The other functions were normal. What treatment should be employed? If we adopt the ligature, it is necessary to attack the external iliac, an operation always very serious when the vessel is diseased. Upon the other hand, alternate compression with two pads was not compatible with the very elevated position of the tumor. M. Michaux decided to make uniform compression upon the pectineal eminence with the little pad suggested by M. Broca. The treatment was commenced on the morning of the 28th November. The compression was intended to be intermittent, that is to say, every four or five hours, the pad was to be loosened, that the patient might have some repose. The next day acute pain forced him to suspend compression for four hours. On the 30th a blister was observed on the compressed shin; suspension of compression for four hours. The pad was placed a little lower down. The compression in this way was suspended and re-established until the 2d December, when from the presence of phlyctena, it was evident that an eschar had formed.

During the four days of treatment, the pain had been acute, particularly in the loins and heel. Inability to sleep had been complete; notwithstanding the employment of opium, the patient was greatly fatigued; the tumor had diminished in size and had become hard; several times when permanent compression had been continued for four or five hours, the pulsations had disappeared. M. Michaux then thought of digital compression; by good fortune there remained a portion of the skin healthy, where the application of the fingers would arrest the pulsations.

The students of the service commenced the task with the greatest enthusiasm—the 5th of December, at 9½ in the morning—the compression was intended to be total. The pulsations of the arteries were energetic, and a considerable force was necessary to close the vessel. Owing to the eschar, compression was very painful. In the afternoon edema, tingling, numbness, stiffness of the limb. At night there was a continuation of these phenomena, cold feet, inability to sleep, great thirst, violent cholic. But the tumor hardened, pulsa tions diminished, the sac was filled with clots, the local ame lioration continued. At nine o'clock in the morning, twenty hours after the commencement of the digital compression, the pulsations and the souffles completely disappeared. The artery did not pulsate below the aneurism, but above the origin of the femoral, the pulsations were energetic. By prudence the compression was continued until two o'clock in the morning, and was then interrupted half an hour to give some repose to the unhappy patient. It was then commenced again, and continued until nine o'clock in the morning, when it was arrested definitely. I stop my narration here, that I may more vividly impress the mind of the lecturer, and pass under silence the details of the general treatment during that period, as well as that during the convalescence. On the 14th of December the eschar was eliminated; it comprised the whole thickness of the skin. Cicatrization was complete on the 16th of January—on the 24th the patient was completely cured. The aneurismal tumor was very hard, and was yet of considerable volume (five centimetres by seven,) but the cure was perfect. If these were the only remarkable observations we had to report, they would be sufficient to recommend digital compression to the profession. Why object to a mode of treatment which cures popliteal aneurisms in five hours, and an aneurism of considerable size of the femoral artery in twenty-four hours? What would have been the fate of the second patient of M. Michaux but for digital compression?

Let us remark that in both cases, the only resource remaining was the ligature—the insufficiency of mechanical compression having been tried. If M. Broca had have had these facts at his disposition when writing his work, he would certainly have modified the chapter in regard to the mode of operating, which we are discussing. Without following step by step the history of digital compression with our learned friend, we hope we will be permitted to discuss some of the propositions which we find in his book. We too, consider digital compression a very "precious resource." M. Broca regards it as a mode that should be reserved for the following cases: 1st, Where the deviation of the member does not permit us to act upon the artery by mechanical means. 2d, Where the excessive irritability of the skin excludes every other mode of compression. But the last of these conditions existed in one and only one of the three preceding cases; the two other cures is an evidence that the field for digital compression should be enlarged. Besides, if we consult the work of M. Broca, we find another remarkable case, that of M. Greatrex. In this the alternate employment of a compressor and the fingers of the patient arrested the pulsations in the tumor in twenty-four hours. In the case of M. Knight, mechanical compression could not be supported more than an hour, while digital compression upon the pubis suffice to cure the patient in forty-eight hours. To crown all, comes the singular observations of the patient submitted to the care of M. Colles, who cured himself by compressing the artery for seven days—the compression was necessarily very imperfect. It is far from my desire to conceal the rather numerous unsuccessful cases by this manner of treatment. But what do we lose if it does fail? Nothing. It is no more than what may result from mechanical compression; it is certainly less unpleasant to the patient, and may be substituted for it with advantage.

For my part, notwithstanding the increasing perfection of the apparatus for mechanical compression, which M. Broca contends makes it rare to meet with a case where digital compression is indicated, I regard it as he—the ideal of the method—and consequently we should reject it only in extreme cases where there is great difficulty of its application. With M. Michaux it would be with great difficulty that I could be made to believe but that in the majority of cases, a sufficient number of aids could be found to assist in an enterprise so noble and so interesting in a scientific point of view. Up to the present time, digital compression has only been adopted in cases where mechanical compression had been tried. My impression is that we should pursue precisely the opposite course, that when compression is indicated, we should commence by compression with the fingers, before having resource to a method less mild. Perhaps it would not be without interest to scan hastily the cases of aneurism in which digital compression had been employed, either alone or in connection with mechanical compression. The number of cases amount to seventeen—ten of which were successful—seven of which were unsuccessful.

Explanations upon the two categories. *

The unsuccessful cases.

Compression was in four cases primitively employed, that is to say, before any other method. 1. Popliteal aneurism. Compression for two days; ligature, (Vanzetti). 2. Popliteal aneurism, only four hours, afterwards mechanical compression for six days (Jameson). 3. Diffused aneurism of the femoral artery, compression seventy-two hours. Apparent cure which, however, was not permanent. Mechanical compression seven days—radical cure. (Parker). 4. Popliteal aneurism. Digital compression three days by patient. Mechanical compression which before was intolerable, became possible—a cure was obtained. (J. Monro.) Twice it was not employed, until mechanical compression was abandoned. 5. Diffused Popliteal aneurism, sixteen days; mechanical compression, ninety-four hours of digital compression, from which the pulsations and souffle disappeared. Success was not permanent; amputation, death. (Nelaton). 6. Popliteal aneurism. For five days mechanical compression was employed, with an imperfect apparatus. Twenty-four hours of digital compres-

*We find in the work of M. Broca the details of the above cases. I give here only a very brief analysis designating them by numbers, and the name of the reporter.

sion. Ligature, amputation, cure. (Norgate). 7. In this case both modes of compression were ineffectual, notwithstanding it was an inguinal aneurism. Compression with the fingers was tried for four days and nights. There was great amelioration. The tourniquet was applied, an eschar formed. The external iliac artery was ligated. (Fox). Of these seven unsuccessful cases, the ligature in three cases was afterwards resorted to, (1, 6, 7,) in two cases amputation became necessary, (5, 6,) one of the cases amputated died. Two of the cases in which the artery was ligated, the patients were cured, (1, 7). In three cases (2, 3, 4,) mechanical compression resulted in a radical cure. In two cases, (3, 5,) digital compression, before it was abandoned, had evidently modified the disease. In another (4) it greatly facilitated the cure by rendering mechanical compression tolerable.

Let us now notice the successful cases. Digital compression was employed primarily in two cases. 8. Popliteal aneurism. Cured in five hours, (Vanzetti.) 9. Patient of M. Colles was cured, who cured himself in seven days, of a Popliteal aneurism by intermittent compression, necessarily very imperfect.

In three cases it succeeded when mechanical compression was abandoned, or inapplicable. 10. Femoral aneurism—mechanical compression for four days. The patient was afterwards cured by digital compression for twenty-four hours, (Michaux.) 11. Popliteal aneurism. Different modes of mechanical compression were tried for a length of time and abandoned. Digital compression was successful in forty-eight hours. 12. Mechanical compression was intolerable. A cure was effected in forty hours by digital compression, (Knight.)

In four cases digital compression was employed in combination with the tourniquet.

This mode, which resembles double and alternate compression, has produced such rapid and decisive results, that it would be well to remark this association of forces, which have been so satisfactory, both to the patient and surgeon. 13 Popliteal aneurism. Tourniquet below the groin; the patient making compression upon the pubis with his fingers. Cured at the end of twenty-four hours, (Greatrex.) 14. Popliteal aneurism.

Digital compression over the pubis, alternating with the compression of Dupuytren. They were employed forty-eight hours—cure. 15. The same lesion and the same method of compression. Eighteen hours sufficed for a cure, (Wood.) 16. The following case, treated by M. Tufferell, is much more complicated. In this case we find associated several modes of compression. A very large popliteal aneurism. Belman's plan of compression was used for two days, and was suspended at the expiration of that time, in consequence of the engorgement of the inguinal glands. Digital compression upon the pubis by the patient and one of his friends; tourniquet lower down, alternating with the compression of the fingers for twenty-four hours. The inguinal pad was afterwards applied; cure at the end of seven days.

In the following case, digital compression was employed conjointly and alternately with mechanical compression and direct compression over the tumor; it is consequently difficult to determine the exact action exercised in the cure. 17. Arterio venous aneurism at the bend of the elbow. Direct compression upon the tumor for several days; afterwards indirect compression with the tourniquet, which could not be supported by the patient. Digital compression by the patient himself was next tried alone. Direct compression upon the tumor was again tried with digital compression made by several aids for twenty-four hours, then abandoned. Afterwards compression was confided alone to the patient, and in a few days the aneurism was cured, (Nelaton.)

We should have more than one remark to make upon these interesting cases, and more particularly as regards the point where the fingers should be applied for compression in popliteal aneurism. Perhaps we would adopt the place of election selected by M. Vanzetti, but we have neither time nor space to enter into this discussion.

From the foregoing we can announce without fear of going too far, the following propositions:

1st. In direct digital compression, continued or even intermittent, executed by the hands of reliable aids, or by the p[illegible]

tient himself, is alone sufficient, without any other aid, either before or after, to cure aneurisms.

2nd. In connection with the tourniquet, and alternating with it, has produced the most simple and rapid cures. As a general thing the cure is rapid when this plan is successful.

3d. By this method, aneurisms have been cured, in which mechanical compression has been impracticable or abandoned. It is much better supported than the latter. Digital compression may be applied upon points where the skin is already inflamed.

4th. This mode of compression is the most efficient and the least painful of all; it permits us to respect the nerves and veins near the arteries, acting alone upon the latter; the skin too may be protected. (Broca, page 807.)

5th. Digital compression may fail; but in such cases it most frequently modifies the condition of the aneurism.

6th. There are sufficient reasons to believe that alone it would have more frequently succeeded, if it had been practised with more perseverance and regularity than has been done in the above mentioned cases.

7th. Up to the present day, there has never resulted any accident from this method.

8th. Applied for the first time with success by Laviard, after an operation for aneurism by the ancient method. Indirect digital compression is then essentially of French origin, and it has not been given even at present all the extension and generalization of which in our opinion it is susceptible.

